# Clinical features of plastic bronchitis in children after congenital heart surgery

**DOI:** 10.1186/s13052-024-01650-9

**Published:** 2024-04-19

**Authors:** Li-Min Zhu, Chun-Xiang Li, Xiao-Lei Gong, Zhuo-Ming Xu, Jin-Long Liu, Hai-Bo Zhang

**Affiliations:** 1 Department of Cardiac Intensive Care Unit, Shanghai Children’ s Medical Center, Shanghai Jiaotong University School of Medicine, 1678 Dongfang Road, 200127 Shanghai, PR China; 2 Institute of Pediatric Translational Medicine, Shanghai Children’ s Medical Center, Shanghai Jiao Tong University School of Medicine, 1678 Dongfang Road, 200127 Shanghai, PR China; 3 Department of Cardiothoracic Surgery, Shanghai Children’ s Medical Center, Shanghai Jiaotong University School of Medicine, 1678 Dongfang Road, 200127 Shanghai, PR China

**Keywords:** Plastic bronchitis, Congenital heart surgery, Fiberoptic bronchoscopy, Airway abnormalities, Children

## Abstract

**Background:**

Plastic bronchitis (PB) can occur in patients who have undergone congenital heart surgery (CHS). This study aimed to investigate the clinical features of PB in children after CHS.

**Methods:**

We conducted a retrospective cohort study using the electronic medical record system. The study population consisted of children diagnosed with PB after bronchoscopy in the cardiac intensive care unit after CHS from May 2016 to October 2021.

**Results:**

A total of 68 children after CHS were finally included in the study (32 in the airway abnormalities group and 36 in the right ventricular dysfunction group). All children were examined and treated with fiberoptic bronchoscopy. Pathogens were detected in the bronchoalveolar lavage fluid of 41 children, including 32 cases in the airway abnormalities group and 9 cases in the right ventricular dysfunction group. All patients were treated with antibiotics, corticosteroids (intravenous or oral), and budesonide inhalation suspension. Children with right ventricular dysfunction underwent pharmacological treatment such as reducing pulmonary arterial pressure. Clinical symptoms improved in 64 children, two of whom were treated with veno-arterial extracorporeal membrane oxygenation (ECMO) due to recurrent PB and disease progression.

**Conclusions:**

Children with airway abnormalities or right ventricular dysfunction after CHS should be alerted to the development of PB. Pharmacological treatment such as anti-infection, corticosteroids, or improvement of right ventricular function is the basis of PB treatment, while fiberoptic bronchoscopy is an essential tool for the diagnosis and treatment of PB. ECMO assistance is a vital salvage treatment for recurrent critically ill PB patients.

## Introduction

Plastic bronchitis (PB) is caused by endogenous tree-like casts or a jelly-like viscous substance blocking the trachea and bronchi, leading to pulmonary consolidation and atelectasis and resulting in partial or complete pulmonary ventilation dysfunction and respiratory distress [1]. This condition often occurs in patients who have undergone congenital heart surgery (CHS), most commonly in children after the Fontan operation (2–3). If no urgent and effective treatment measures are taken, the condition often deteriorates rapidly, leading to respiratory failure and even death [4]. The key to treating PB is timely diagnosis and improvement of respiratory failure, removing substances that cause bronchial obstruction, and inhibiting cast formation [5].

Recently, due to the popularization of fiberoptic bronchoscopy (FOB) in the cardiac intensive care unit (CICU), clinicians have a further understanding of the diagnosis and treatment of PB in children after CHS. However, PB’s clinical characteristics, treatment, and prognosis still need to be further studied. This study aims to review the clinical data of children with PB after CHS and to explore the clinical characteristics of such children to provide a reference for the diagnosis and treatment of PB after CHS.

## Materials and methods

### Patients

We conducted a retrospective cohort study of patients usingthe electronic medical record system of Shanghai Children’s Medical Center from May 2016 to October 2021. Patient data, including clinical characteristics, laboratory findings, imaging, and pharmacological management, were collected at admission. Children under 18 years of age who met the diagnostic criteria for PB after CHS were enrolled in the study. This study was approved by the Ethics Committee of Shanghai Children’s Medical Center (SCMCIRB-K2017018). Patients with a history of foreign body inhalation and incomplete medical records were excluded.

### Definitions

The diagnosis of PB was based on the following: (1) FOB findings: respiratory mucosa congestion, edema and excessive secretion; the bronchial lumen was occluded by inflammatory bronchial casts and showed a “dendritic” structure; (2) Histopathology findings: Inflammatory bronchial casts consisted of inflammatory cells (mainly eosinophils and neutrophils) and shed epithelial cells [6]. In 1997, Seear et al. developed a classification system for PB by allocating the cases into two types. The type 1, inflammatory casts composed of fibrin and dense inflammatory infiltrate, associated with asthma or respiratory infection. The type 2, acellular casts composed mostly of mucin, associated with cyanotic congenital heart diseases (CHD) [7].

### FOB procedure

Indications for FOB are as follows: (1) Children with spontaneous breathing showed shortness of breath, wheezing, cyanosis and dyspnea. (2) Mechanically ventilated children showed increased airway pressure, hypoxemia, hypercapnia and unconsciousness. Gastric contents were emptied through a nasogastric tube. Propofol (1–2 mg/kg) was used for anesthesia induction. In addition, the dosage can be appropriately increased according to the degree of sedation of children during the operation. The children with spontaneous breathing were treated with a 2% lidocaine hydrochloride injection in the nasal cavity for topical anesthesia. After successful anesthesia, fiber bronchoscope Olympus BF-XP290F (exterior diameter of the front end: 2.8 mm, inner diameter of the tube: 1.2 mm) or BF-P290F (external diameter of the front end: 4.0 mm, internal diameter of the tube: 2.0 mm) was selected and placed through the nostril or side hole of the Y-shaped tube of the endotracheal intubation. The nasal cavity, epiglottis, glottis, trachea, tracheal carina, and bronchi were detected in turn. Negative pressure was used to suck foamy or jelly-like substances. For the jelly-like obstructions that were difficult to be sucked directly by negative pressure, 0.9% normal saline 2–4 ml could be repeatedly injected to loosen the obstruction before suction, and 0.9% normal saline 5–10 ml was injected into the lesion site for bronchoalveolar lavage (BAL). If casts could not be sucked, they could be removed using biopsy forceps. At the end of the operation, 3–5 ml of the bronchoalveolar lavage fluid (BALF) was sent for pathogenic examination, and the casts were sent for pathological examination.

After the operation, the children were kept supine and fasted for at least 2 h. The breathing, airway pressure and blood oxygen saturation were closely observed and the chest X-ray was reexamined. Some children need to repeat FOB according to their condition. In addition, children were tested for serum Immunoglobulin E and allergens when PB was suspected.

## Results

### Baseline characteristics

**Fig. 1:**
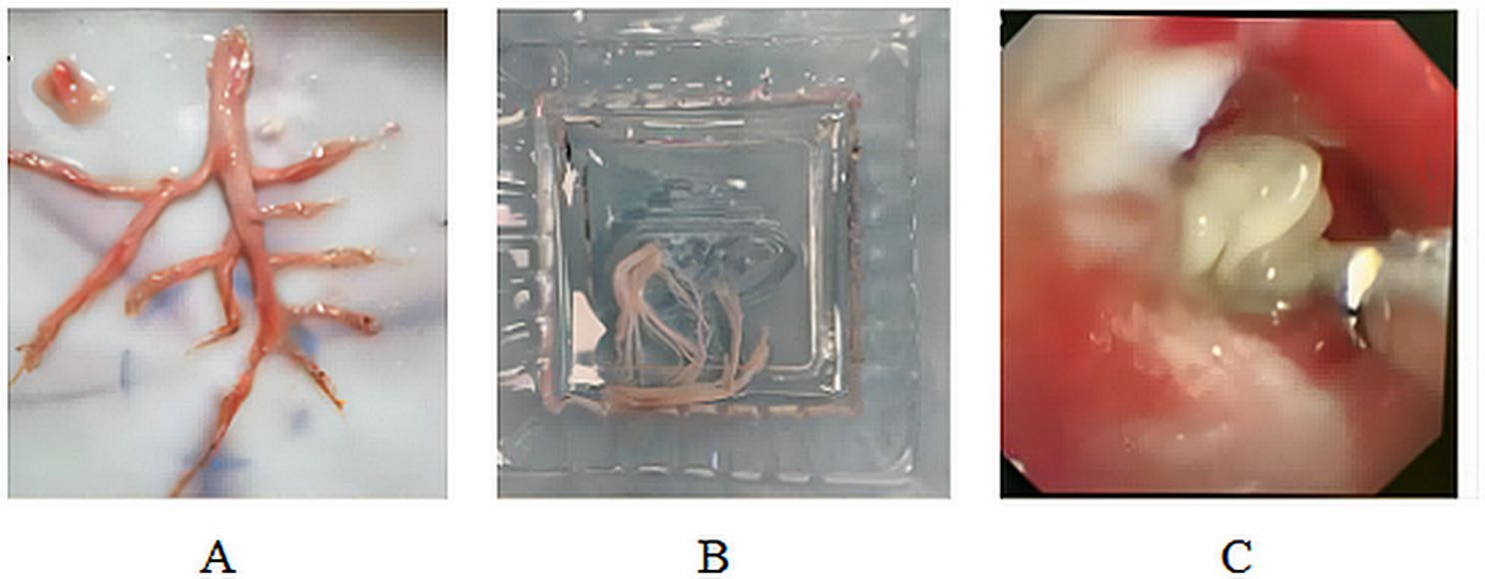
Casts **A**: The tree-like cast from a child after Fontan operation **B**: The casts in children with congenital tracheal stenosis sucked by fiberoptic bronchoscopy **C**: fiberoptic bronchoscopy

**Fig. 2:**
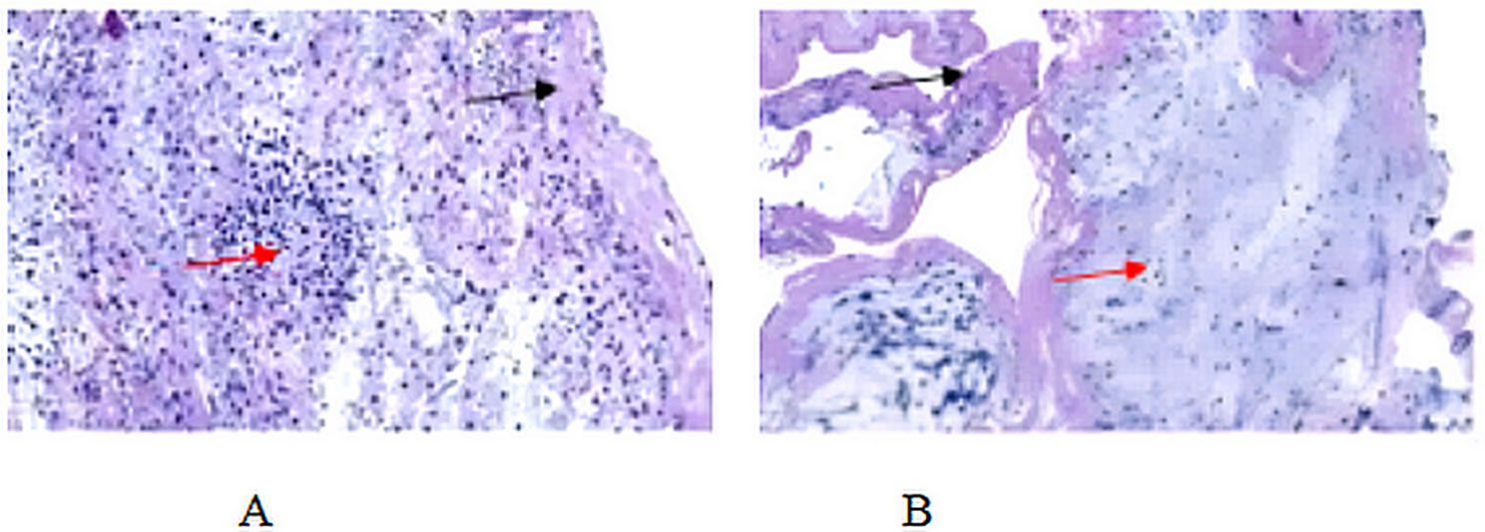
Pathology **A**: pathological manifestations of the airway abnormalities group. The Red arrows point to neutrophils and black arrows point to cellulose **B**: pathological manifestations of the right ventricular dysfunction group. The Red arrows point to neutrophils and black arrows point to cellulose

A total of 70 children were screened from May 2016 to October 2021, of whom 2 did not meet the inclusion criteria due to incomplete data. Ultimately, 68 children were included in the study. Children were divided into the airway abnormalities group (*n* = 32) and right ventricular dysfunction group (*n* = 36) according to the cause of PB formation. The baseline characteristics in the two groups are shown in Table 1. Cord-like casts or tree-like casts were detected in all children (Fig. 1). Neutrophil infiltration surrounded by fibrin was seen on the pathological smear in the airway abnormalities group. A small number of neutrophils and fibrin were observed in the pathological smear of the right ventricular dysfunction group, which was significantly less than that of the airway abnormalities group (Fig. 2).

**Table 1:** Baseline characteristics

Group	Surgery	Case (n)	Gender (M/F)	Age (month)	Weight (kg)	CVP (cmH2O)	CPB (min)	IMV (day)
Airway abnormalities	Vascular ring correction	12	5/7	5	5.5	6	0	1
	PAS correction + tracheoplasty	20	14/6	10	9	7	108	3
Right ventricular dysfunction	Fontan	15	8/7	38	13	15	95	3
	Glenn	6	3/3	16	10.5	13	92	2
	PA/VSD correction	6	4/2	20	12	14	126	4
	Tricuspid valvuloplasty	3	1/2	24	10.5	12	45	5
	Glenn + TAPVC correction	6	4/2	6	6.5	14	106	3

The median was used for age, weight, CVP, duration of CBP, and duration of IMV; PAS: pulmonary artery sling; PA/VSD: Pulmonary atresia with ventricular septal defect; TAPVC: total anomalous pulmonary venous connection; M/F: male/female; CVP: central venous pressure; CPB: cardiopulmonary bypass; IMV: invasive mechanical ventilation

The airway abnormalities group included 20 cases of PAS combined with congenital tracheal stenosis (CTS) and 12 cases of vascular ring correction. The right ventricular dysfunction group included 15 cases of Fontan operation, 6 cases of Glenn operation, 6 cases of single ventricle with complete atrioventricular septal defect and total anomalous pulmonary venous connection (SV/CAVC/TAPVC) which underwent Glenn and TAPVC correction, and 3 cases of tricuspid valvoplasty and 6 cases correction of pulmonary atresia with ventricular septal defect (PA/VSD). Among the group of right ventricular dysfunction, there were 9 cases of biventricular repair procedure and 27 cases of palliative procedure for functional single ventricle. All the patients presented the symptoms of systemic venous congestion such as edema, ascites and pleural effusion, for patients underwent biventricular repair, the echocardiography showed the hypertrophy or dilation of right ventricle. There were 11 cases of heterotaxia syndrome, 6 cases of hypoplastic of right ventricle, 2 cases of hypoplastic left heart syndrome and 8 cases of complex congenital heart defect incapable of undergoing biventricular repair procedure in the patients with single ventricular physiology.

### Pathogenic findings

**Fig. 3:**
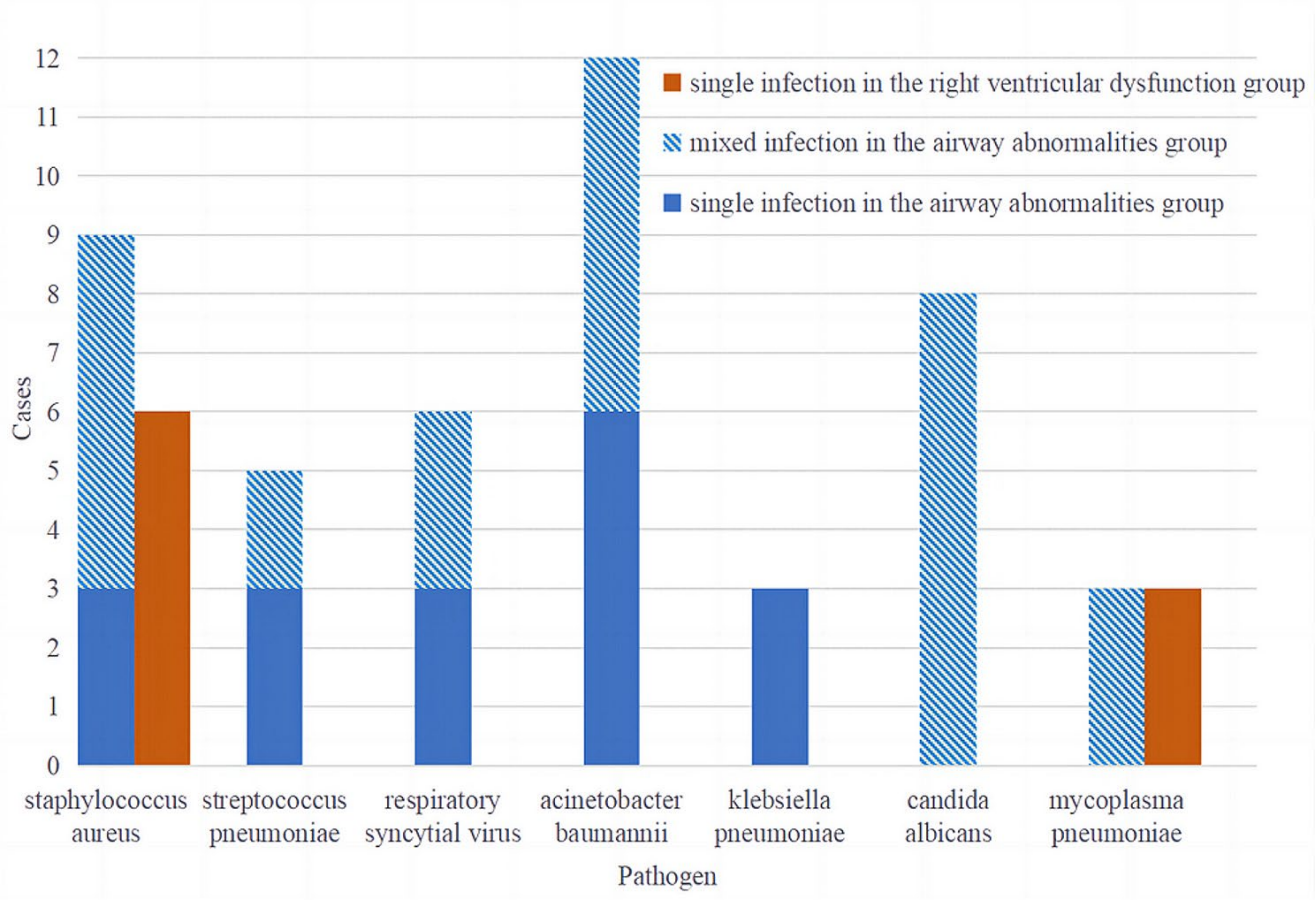
Etiological examination results

Of the 68 children, 42 received one FOB, 18 received two FOBs and 8 received more than two FOBs. The BALF of all children was sent for pathogenic examination and different pathogens were detected in 41 children (Fig. 3). Pathogens were found in 32 cases in the airway abnormalities group. Among them, 18 cases were single pathogen infections, including 3 cases of Staphylococcus aureus, 3 cases of Streptococcus pneumoniae, 3 cases of Respiratory syncytial virus, 3 cases of Klebsiella pneumoniae and 6 cases of Acinetobacter baumannii; 14 cases were mixed infections, including 3 cases of Staphylococcus aureus and Acinetobacter baumannii, 3 cases of Staphylococcus aureus and Candida albicans, 3 cases of Respiratory syncytial virus and Candida albicans, 3 case of Mycoplasma pneumoniae and Acinetobacter baumannii and 2 cases of Streptococcus pneumoniae and Candida albicans. There were 9 cases of single pathogen infection in the right ventricular dysfunction group, including 6 cases of Staphylococcus aureus and 3 cases of Mycoplasma pneumoniae.

### Treatment

Pharmacological treatment was shown in Table 2. All patients received at least one type of antibiotic and the antibiotic regimen was adjusted based on pathogenic findings. All patients were treated with intravenous or oral corticosteroids and budesonide inhalation suspension. The frequency of aerosol therapy varied from once an hour to once every 8 h according to the severity of clinical symptoms. Eighteen patients had recurrent casts and were treated with urokinase-type plasminogen activator (uPA) combined with chymotrypsin for aerosol therapy. No airway bleeding, aspiration or allergy, or other complications occurred during the aerosol therapy, and the clinical symptoms were relieved after treatment. Twenty-seven children in the right ventricular dysfunction group were treated with continuous intravenous injections of treprostinil (10–50 ng/kg.min) combined with oral sildenafil (0.5-1 mg/kg tid) or bosentan (2 mg/kg bid) to reduce pulmonary vascular resistance.

**Table 2:** Medication

Medication	Airway abnormalities group	Right ventricular dysfunction group
Acetylcysteine solution combined with budesonide suspension for aerosol therapy	32	36
Urokinase-type plasminogen activator combined with chymotrypsin for aerosol therapy	15	3
Intravenous or oral glucocorticoids	32	36
Continuous intravenous injection treprostinil combined with oral sildenafil or bosentan	0	27

### Clinical outcomes

The clinical symptoms of 64 children were improved. Two children after PAS correction and tracheoplasty were supported by venous-arterial extracorporeal membrane oxygenation (V-A ECMO) because the repeated casts completely blocked the trachea and bronchi and the condition continued to worsen. The FOB was performed 6 times during ECMO support. Anti-infection therapy, aerosol therapy, and other support therapy were used and the two patients successfully weaned from ECMO after 5 days and 7 days, respectively. After the Fontan operation, a child was admitted to the hospital because he coughed up tree-like casts and was treated with fiberoptic bronchoscopy to remove the casts and BALF twice (Fig. 1A). Anti-infection, reduction of pulmonary vascular resistance, and other routine treatments were given, and the patient was discharged at 2 weeks. Four children died due to co-morbid severe pulmonary infections.

There were 7 cases of specific allergies and increased plasma Immunoglobulin E in the airway abnormalities group, including 5 cases of PAS + CTS, 2 cases of the vascular ring. There were 3 cases of chylothorax in the right ventricular dysfunction group.

## Discussions

In this study, we summarized the etiology, clinical manifestations, and treatment of 68 children with PB. We found that children with airway abnormalities or right ventricular dysfunction after CHS were prone to PB. Anti-infection, corticosteroids, or improving right ventricular function are helpful in treating PB, and FOB is an essential tool for diagnosing and treating PB. For recurrent critically ill PB patients, ECMO assistance is a vital salvage treatment.

During the research span of more than 5 years, we screened 13,752 children with CHD. Of the 68 children with PB, 32 had airway abnormalities and 38 had right heart dysfunction, so we should be alert to PB for children with these abnormalities after CHS. The pathology of PB in the airway abnormalities group was characterized by neutrophil infiltration surrounded by fibrin, which is consistent with the performance of type 1 in the PB classification [7]. Studies have shown that infection and allergy are the main factors related to the pathogenesis of PB in type 1. Inflammatory stimulation causes tracheal mucosa injury, necrosis, increased mucus secretion and easily forms mucus plugs to block the airway and accelerate cast formation [8]. In addition, normal respiratory florae are generally not pathogenic. However, in the absence of good clearance mechanisms and effective innate or acquired immune responses, these normal airway florae can invade the lower respiratory tract and cause disease [9]. In this study, the BALF of all children was sent for pathogenic examination and the incidence of infection was up to 100% in the airway abnormality group. The reason for this may be the combination of the airway abnormalities group with tracheal stenosis, tracheomalacia or tracheospasm, which interferes with normal airway clearance and immune function. Airway surgery can disrupt the airway’s filtering and cleaning ability, and pathogens may be more likely to cause airway inflammation and promote the development of PB. Therefore, we believe that, especially in children with airway abnormalities after CHS, more attention should be paid to the prevention and treatment of infections to avoid or improve the occurrence and development of PB. In addition, there were 7 children with a special allergic constitution, which may be a risk factor of type 1 PB in these children.

The present study found that the pathology of PB with right ventricular dysfunction showed a small number of neutrophils and fibrin infiltration, which was consistent with the pathology of type 2 PB [7]. Studies have shown that the occurrence and development of type 2 PB are related to abnormal lymphatic circulation (10–11). Children after Fontan or Glenn surgery may be due to the high flow of pulmonary blood vessels or increased pulmonary vascular resistance, which leads to the increase of intrapulmonary vascular exudation and the aggravation of a load of intrapulmonary lymphatic circulation (12–13). Our study also confirmed that some children with right ventricular dysfunction were treated by reducing pulmonary vascular resistance and improving right ventricular function, and their clinical symptoms improved and they were discharged from the hospital. Normally, lymphatic flow is centered and regulated by valves, but dysfunction of lymphatic vessels or injury to the thoracic duct can lead to abnormal blood flow patterns [14–16]. In our study, there were 3 cases of chylothorax in the right ventricular dysfunction group, and 2 cases of children with thoracic duct dilatation and valvular insufficiency were caused by increased flow in the thoracic duct after Fontan and PA/VSD correction surgery, respectively. The other patient had chylothorax after Glenn’s operation.

Pharmacological treatment is an important treatment for PB. Several studies have pointed out that respiratory infection is closely related to the occurrence of PB (17–18). In this study, the BALF of all children was sent for pathogenic examination and different pathogens were detected in 41 children. The incidence of infection up to about 60%. Antibiotics is the primary treatment and should be adjusted in time according to the pathogenic examination results of BALF. Another study pointed out that corticosteroids are therapeutic in PB by mediating the anti-inflammatory effect on eosinophil infiltration [19]. In this study, all children were treated with intravenous or oral corticosteroids, combined with budesonide suspension for aerosol inhalation and most children achieved good treatment results. Additionally, studies suggested that inhaled tissue prothrombin activator (tPA) and uPA can be used to treat PB (20–21). In this study, 18 children with recurrent casts were treated with uPA combined with chymotrypsin inhalation, all of them were relieved and no side effects were found. In addition to symptomatic treatment, active treatment of the primary disease also needs to be strengthened. For patients with right ventricular dysfunction, reducing pulmonary vascular resistance and increasing ventricular filling pressure may help prevent PB (22–23).

Nonpharmacological treatment includes postural drainage, pulmonary physiotherapy and mechanical ventilation. Among them, FOB to remove foreign bodies and plaster in the bronchi is the most common and effective treatment method [5]. All children in this study underwent FOB and BAL to suck the casts causing trachea and bronchi obstruction. Meanwhile, the pathogen test results of BALF can help target the application of antibiotics. In our study, 18 children received two FOBs and 8 received more than two. After FOB, the clinical symptoms of these children were significantly relieved.

Two patients in this study were supported by V-A ECMO after PAS correction and tracheoplasty due to continuous deterioration of the trachea and bronchi with repeated casts. During ECMO assistance, we used anti-infection, nebulized inhalation of acetylcysteine, budesonide, uPA and chymotrypsin, combined with fiberoptic bronchoscopy and the two patients were successfully weaned from ECMO finally. This confirms the effectiveness of pharmacological and nonpharmacological treatment combinations in critically ill children with PB.

### Limitations

There were several limitations to this study. Firstly, the retrospective nature of our study suggests that our findings may be subject to selection bias. Moreover, due to the limited number of cases, the number and types of diseases included in this study are limited. Finally, Since the patients in this study were children after CHS, the results cannot be extrapolated to children with other diseases.

## Conclusions

Children with airway abnormalities or right ventricular dysfunction after CHS should be alerted to the development of PB. Pharmacological treatment such as anti-infection, corticosteroids, or improvement of right ventricular function is the basis of PB treatment, while fiberoptic bronchoscopy is an essential tool for the diagnosis and treatment of PB. ECMO assistance is a vital salvage treatment for recurrent critically ill PB patients.

## Data Availability

The datasets used or analyzed during the current study are available from the corresponding author upon reasonable request.
